# Very-Low-Absorbable Geraniol for the Treatment of Irritable Bowel Syndrome: A “Real-World” Open-Label Study on 1585 Patients

**DOI:** 10.3390/nu17020328

**Published:** 2025-01-17

**Authors:** Chiara Ricci, Ilaria Maria Saracino, Maria Chiara Valerii, Renato Spigarelli, Irene Bellocchio, Enzo Spisni

**Affiliations:** 1 Gastroenterology Unit, ASST Spedali Civili di Brescia, University of Brescia, Piazza del Mercato 15, 25121 Brescia, Italy; chiara.ricci@unibs.it; 2 Microbiology Unit, IRCCS, Azienda Ospedaliero-Universitaria di Bologna, University of Bologna, Via Massarenti 9, 40138 Bologna, Italy; ilariamaria.saracino@studio.unibo.it; 3 Department of Biological, Geological and Environmental Sciences, University of Bologna, Via Selmi 3, 40126 Bologna, Italy; renato.spigarelli@studio.unibo.it (R.S.); irene.bellocchio2@studio.unibo.it (I.B.); enzo.spisni@unibo.it (E.S.)

**Keywords:** irritable bowel syndrome, geraniol, dietary supplement, IBS-SSS

## Abstract

Objective: The objective of this study was to evaluate the efficacy of a very-low-absorbable geraniol formulation, administered as a food supplement, in patients with irritable bowel syndrome (IBS) in a real-world setting in Italy. Methods: This open-label study was conducted in Italy on patients diagnosed with IBS and treated for 4 weeks with 240 mg/day of Palmarosa essential oil, absorbed on 960 mg of ginger root powder to obtain a very-low-absorbable geraniol formulation. Baseline characteristics, including demographic and symptoms were recorded using the IBS Severity Scoring System (IBS-SSS). After 28 ± 7 days, the patients were asked to complete the IBS-SSS questionnaire again. The primary objective was to confirm the effects of a very-low-absorbable geraniol formulation on self-reported symptoms of IBS and the quality of life of affected individuals. The secondary objective was to confirm the effect of the treatment on the different IBS subtypes. Results: A total of 1585 patients were included in the study, with a mean age of 44.8 years and 56.4% women. Following the 4-week supplementation period, significant decreases were observed in the patients’ IBS-SSS (−67.9%) and all the primary IBS symptoms, such as abdominal distention (−82.3%), unsatisfaction with bowel habits (−46.2%), and interference with quality of life (QoL) (−64.9%) (all *p* < 0.01). The patients’ stool type improved significantly. Treatment was effective in all IBS subtypes. Conclusions: Treatment with very-low-adsorbable geraniol food supplement was associated with improvements in symptoms and bowel habits in all IBS subtypes in a real-world setting in Italy. These findings support the use of geraniol as an effective option for patients with IBS regardless of the disease subtype.

## 1. Introduction

Irritable bowel syndrome is currently the most common functional gastrointestinal disorder [1,2]. It is a chronic relapsing condition that, in the absence of any other causal disease, can be defined by the presence of certain symptoms without known anatomical explanations. The main symptoms include abdominal distension (the objective feeling of fullness/bloating), abdominal pain, discomfort, and, especially, altered bowel habits (constipation, diarrhea, or both) [2,3]. According to the Rome IV diagnostic criteria, IBS can be classified into four subtypes based on the predominant bowel pattern: IBS-C with constipation; IBS-D with diarrhea; IBS-M mixed with alternating constipation; and IBS-U unclassified in the previous categories [4]. However, it is possible for the same patient to experience overlapping subtypes or for bowel patterns to change over time, leading to a shift from one subtype to another [2].

Observational studies report a substantial (≥20%) association with other functional gastrointestinal disorders (such as dyspepsia, heartburn, gastroesophageal reflux disease, nausea, etc.) [5] and non-gastrointestinal disorders (such as fibromyalgia syndrome, chronic fatigue syndrome, migraines, eating disorders, food intolerances, etc.) [6]. Most of these studies also note the presence of psychiatric comorbidities (like anxiety, depression, somatization, or neurosis). In fact, the entire pathological framework of IBS has been included as a “somatic symptom disorder” in the Diagnostic and Statistical Manual of Mental Disorders, 5th edition [7], and also in psychiatric or psychosomatic clinical practice [8]. Although IBS is considered a benign condition, it often significantly impairs quality of life and social functioning [9,10]; indeed, it is the second leading cause of work absenteeism and has a significant economic impact on the National Health System. It is estimated that around 25–50% of all gastroenterological consultations are conducted for the management of this disorder [11].

Epidemiological studies have shown that the prevalence of IBS varies across geographical areas; although it ranges between 5% and 10% in most regions, it should be considered that estimates between studies may vary, partly because of methodological heterogeneity (related to the use of Rome III or Rome IV criteria) [12,13]. Women are two to four times more likely to develop IBS than men [2] and tend to suffer more from abdominal pain and constipation, rather than diarrhea, which is more common in the male population [14]. Finally, the prevalence of IBS decreases with age; many cases develop in early childhood, with the majority occurring in patients under 45 years of age [15].

The etiology of the disease is complex and not yet completely clear. It is certainly a multifactorial condition, with multiple contributing factors [16] such as genetic predisposition or family history, psychosocial and emotional factors (e.g., stress), diet and food intolerances, alterations in intestinal motility, increased intestinal permeability, visceral hypersensitivity, intestinal dysbiosis, enteric infections, silent chronic inflammation, abnormal immune response, hormonal variations, etc., [17,18]. It has also been observed that the chronic use of certain medications, such as anti-inflammatories and antibiotics, can worsen symptoms or alter individual microbiota, promoting the development of IBS [4].

Given the complex pathophysiology and the absence of biological markers, diagnosis is essentially clinical, and there is no treatment that works for all patients. Symptom relief is currently the primary goal [1]: treatment is chosen based on the specific clinical situation and requires a multidisciplinary approach [2]. Traditionally, IBS therapies have been based on the use of over-the-counter medications aimed at regulating bowel habits for their widespread availability, low cost, and high level of safety [15]. However, some of these drugs cannot be administered for long periods, have contraindications or side effects, and offer few benefits [2]. In recent years, lifestyle interventions (diet and physical activity) and alternative therapies to conventional medicine (like phytotherapy) have become increasingly important first-line treatment options [15].

Several studies have also shown that the microbiota of patients with IBS presents alterations, such as lower microbial diversity, associated with a lower amount of *Lactobacillus*, *Bifidobacterium*, and *Bacteroides* spp., and a higher amount of *Pseudomonadota* (formerly known as *Proteobacteria*), as well as general phylogenetic and functional instability [19,20,21,22]. These findings have led to the belief that probiotics could be used for the management of IBS due to their effects on improving the intestinal barrier, inhibiting pathogen adhesion to the intestinal mucosa, and modulating metabolic and nutritional profiles at the intestinal lumen level [19,20,21,23,24]. However, the results of studies in which IBS patients were treated with probiotics have not confirmed the expected outcomes [25], and the results largely depend on the IBS subtype [26].

Current knowledge does not allow us to establish if the alteration of the microbiota associated with IBS plays a causal role as a pro-inflammatory factor or is, instead, a consequence of local inflammation. Certainly, dysbiosis plays a role in the pathophysiology of the disease and, probably, also in the persistence of intestinal symptoms.

Essential oils have proven useful in modulating the pathophysiology of the colon and managing intestinal diseases, considering the role of inflammation, immune activation, oxidative stress, and dysbiosis in their development [27].

Geraniol, specifically, is a non-toxic compound [28], classified as GRAS (Generally Recognized As Safe) by the FDA (Food and Drug Administration). Its pharmacological properties, such as selective antimicrobial [29], anti-inflammatory [30], antioxidant [31], and neuroprotective properties, have long been known; it has also been pointed out that geraniol possesses anti-nociceptive activities, probably related to the modulation of glutamatergic neurotransmission [32].

In an in vivo study on a mouse model with Dextran Sulfate Sodium (DSS)-induced colitis, orally administered low-adsorbable geraniol (30 and 120 mg/kg per day) reduced the expression of Cyclooxygenase-2 (COX-2) in the intestinal wall and significantly improved colitis and dysbiosis [33].

The same low-adsorbable geraniol formulation, administered orally (8 mg/kg per day), has been shown to be a potent modulator of the microbiota in a pilot study conducted on patients with IBS, reducing the IBS-Visual Analog Scale (IBS-VAS) scores and improving the quality of life for these patients [34].

Free geraniol has an absolute bioavailability of 92%, which drops to 50% in low absorbable forms (microencapsulated in sunflower lecithin) used in mice colitis and in the pilot study by Rizzello and coworkers [34]. The adsorption of geraniol on vegetable fiber gives rise to a very-low-absorbable formulation of geraniol, reducing its absolute bioavailability to 16% and thus delivering about 85% of geraniol to the colon [35].

Ginger (*Zingiber officinale*) rhizome fiber was selected as the vegetable “carrier” for geraniol in a double-blind, randomized, placebo-controlled clinical trial on 56 IBS patients [36]. The results showed efficacy in reducing the severity of the disease and symptoms, assessed with the IBS-Severity Scoring System (IBS-SSS), and improving the composition of the microbiota, especially in the IBS-M subtype. Therefore, this dietary supplement based on geraniol adsorbed on ginger rhizome fiber could become a valid and interesting alternative to the use of over-the-counter medications for managing IBS, which can be safely taken even for long periods of time.

The study by Ricci and coauthors [36] had some limitations, such as the small number of enrolled patients and the prevalence of the IBS-M subtype, making it difficult to extend the results to other IBS subtypes. Additionally, the trial was designed and registered before the release of the Rome IV diagnostic criteria, so the use of Rome III criteria might have introduced a bias in selecting the study population. Hence, in the present research, we decided to verify, through a “real-life” open-label study, the effects of a dietary supplement based on Palmarosa (*Cymbopogon martinii*) essential oil with a high geraniol content, adsorbed on ginger vegetable fiber, on the intestinal symptoms and habits of a much larger number of patients with irritable bowel syndrome.

## 2. Materials and Methods

### 2.1. Study Design and Population

The study was an interventional multicenter clinical trial conducted in an open-label manner with dietary supplements. The total duration of the study was 9 months, and the enrollment period was 8 months. Each patient in the study was administered a food supplement based on Palmarosa (*Cymbopogon martinii*) essential oil titrated in geraniol and adsorbed on Ginger (*Zingiber officinale*) vegetable fiber for 30 days. The study was approved by the Independent Ethics Committee for Nonpharmacological Clinical Investigations on 7 February 2024. The patients were informed of the full nature and purpose of the study and provided written informed consent before entering the trial. The sites involved in enrolment were private, and the study was conducted in conformity with the principles of the Declaration of Helsinki and Good Clinical Practice. The study population consisted of patients diagnosed with IBS, enrolled by 31 private Italian gastroenterologists. Inclusion criteria were age between 18 and 65 years; signed informed consent; diagnosis of IBS according to the Rome IV criteria; and body weight between 48 kg and 104 kg, with a BMI < 27. Exclusion criteria were known or suspected hypersensitivity to Palmarosa essential oil, Ginger, or any excipients contained in the dietary supplement used for the treatment; diagnosis of IBD, celiac disease, or other severe systemic diseases; severe concomitant diseases that, in the investigator’s opinion, contraindicate the patient’s participation in the study; lactose intolerance or confirmed food allergies; use of steroidal anti-inflammatory drugs, antibiotics, or supplements and/or functional foods containing pre-/probiotics in the month preceding enrollment; and use of experimental drugs in the two months preceding enrollment.

The patients were diagnosed according to Rome IV criteria in case of recurrent abdominal pain, which began at least 6 months earlier and was present during the previous 3 months for at least 1 day per week. All of this was associated with a change in the frequency of evacuations (constipation, diarrhea, or alternation) and a change in stool consistency [18]. The score was calculated using the validated IBS-SSS questionnaire [37] (Supplementary Materials, Figure S1). This study was registered in the ISRCTN registry, ID: ISRCTN12928913.

The patients were divided into 4 subtypes:

1. IBS-C with constipation (>25% hard stools and <25% soft stools).
2. IBS-D with diarrhea (>25% soft stools and <25% hard stools).
3. IBS-M with mixed bowel habits (>25% soft stools and >25% hard stools).
4. IBS-U not classifiable into any of the previous subtypes (<25% soft stools and <25% hard stools).

The Bristol stool chart was used to recognize stool types, with values ranging from 1 (hard, lumpy stools) to 7 (soft, watery stools) [38].

### 2.2. Treatment

The food supplement (Eurekol™) was provided by Diadema Farmaceutici S.R.L. (Pisa, Italy) to each investigator and properly stored in a cool, dry place away from heat sources. Each package contained 30 gastro-resistant capsules composed of 600 mg of BIOintestil^TM^ [Ginger (*Zingiber officinale* Rosc.—rhizome), Palmarosa essential oil (*Cymbopogon Martinii* Will. Watson—aetheroleum)], hydroxypropyl cellulose, microcrystalline cellulose, silicon dioxide, calcium phosphate, magnesium stearate, and white gastro-resistant coating (without TiO_2_) [shellac, hydroxypropyl methylcellulose, talc, calcium carbonate, polyethylene glycol, glycerol]. The daily dosage used in the present trial was 2 capsules/day, corresponding to 204 mg of geraniol. This dosage was very similar to the average dose used in our previous study [36] of 210 mg/day.

Table 1 reports the quantitative composition of actives for 2 capsules.

Each investigator assigned each enrolled subject two packages of Eurekol™, sufficient to cover the recommended daily dose for the 30 days of the planned treatment period. Each patient was asked to return the supplement packs at the end of the experimental process so that each investigator could proceed with the accounting in order to assess the compliance (calculated according to the ratio of the number of capsules taken to the number of capsules theoretically expected).

Patients who consumed less than 90% of the capsules were considered “drop-outs”, and those who did not undergo follow-up within 7 days of the predetermined date were considered “lost to follow-up”. Participation in the study could be interrupted at any time if it was deemed beneficial for the patient’s health or at the patient’s own discretion.

### 2.3. Aims of the Study

The primary objective of the study was to confirm the effect of a very-low-absorbable geraniol formulation (Eurekol^TM^ based on BIOintestil^TM^) on self-reported symptoms of IBS and the quality of life of affected individuals in a large sample of patients.

The secondary objectives were to confirm the effect of treatment with a very-low-absorbable geraniol formulation on the different IBS subtypes.

### 2.4. Study-Specific Visits

#### 2.4.1. Visit 1—Baseline

The investigator, after presenting the study to the selected patient and obtaining informed consent, assessed the inclusion and exclusion criteria to confirm eligibility. For eligible subjects, a medical history and a clinical examination were performed as per standard clinical practice, during which some information, in particular about the clinical history, symptoms, and any concomitant conditions and therapies, was collected. Every patient was asked to complete the IBS-SSS questionnaire and then received two packages of Eurekol^TM^. The patients were enrolled sequentially, and the objective of the geraniol treatment was clearly explained to each patient during the inclusion visit.

#### 2.4.2. Visit 2—Week 4 (±7 Days)

The inclusion and exclusion criteria were verified again to reconfirm the patient’s eligibility. A recent medical history and a second clinical examination were performed as per standard clinical practice, during which some information, in particular about the patient’s symptoms, any changes since the previous visit, adverse events, and therapies taken, was collected. Every patient was asked to complete the IBS-SSS questionnaire again and return the packages of Eurekol™. Therefore, the investigator proceeded with the evaluation of compliance.

### 2.5. Statistical Analysis

All analyses were performed using the SPSS statistical software version 23 (SPSS Inc., Chicago, IL, USA).

The data are presented for the per-protocol population, meaning all patients who completed the study were in full compliance with the protocol and without any major deviations. Continuous variables are expressed as mean and standard deviation, frequencies as number, %, and 95% confidence interval. The paired Student *t*-test was used to compare continuous variables before and after the treatment. For pre-post frequencies, McNemar’s Chi-square test was used, or McNemar’s exact test correction when the contingency table contained at least one box with a frequency ≤ 5. A *p*-value ≤ 0.05 or ≤0.01 was considered statistically significant.

## 3. Results

### 3.1. Population

A total of 1736 patients met the inclusion criteria and were enrolled for the study. Eighty-two patients were lost to follow-up because they did not undergo V2 within the protocol timeframe. A total of 69 patients were considered drop-outs for not achieving full compliance (at least 90% of the capsules consumed); among them, only 34 patients (2%) stopped taking the supplement due to adverse events potentially related to the intake of geraniol (unspecified gastric symptoms).

Data from 1585 patients were available (mean age 44.8 ± 13.7 years). Among them, 691 (43.60%) were males (mean age 44.7 years), and 894 (56.40%) were females (mean age 44.9 years). A total of 473 patients were diagnosed with IBS-C, 484 with IBS-D, and 474 with IBS-M. The remaining 154 subjects who could not be included in any of the previous three categories were considered IBS-U. Table 2 and Table 3 show the characteristics of the population stratified by gender, by type of diagnosed IBS, and by age group.

As shown in the tables, most patients were women, the most diagnosed IBS subtype was diarrheal, and the most represented age group ranged between 41 and 50 years old.

### 3.2. Results from the Analysis of the IBS-SSS Questionnaires

The IBS-SSS Score was calculated as described in the Supplementary Materials (Figure S1) for each patient at V1 and V2. Table 4 reports the main results of the IBS-SSS questionnaire and the % of responders.

**Figure 1:**
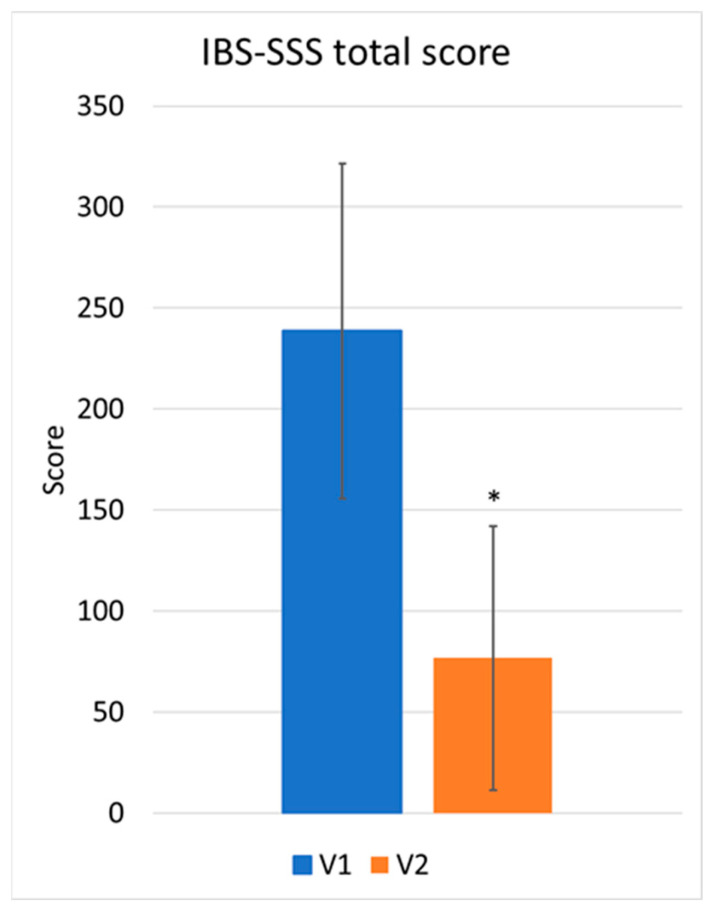
IBS-SSS total score at V1 and V2 (mean and S.D.) * *p* < 0.01.

Statistical analyses identified significant differences in the participants’ symptomatology between V1 and V2. Figure 1 demonstrates a highly significant reduction, with *p*-value < 0.01, in the total score after the 30 days of treatment, with the mean value dropping from 238.66 ± 82.91 to 76.69 ± 65.35.

**Figure 2:**
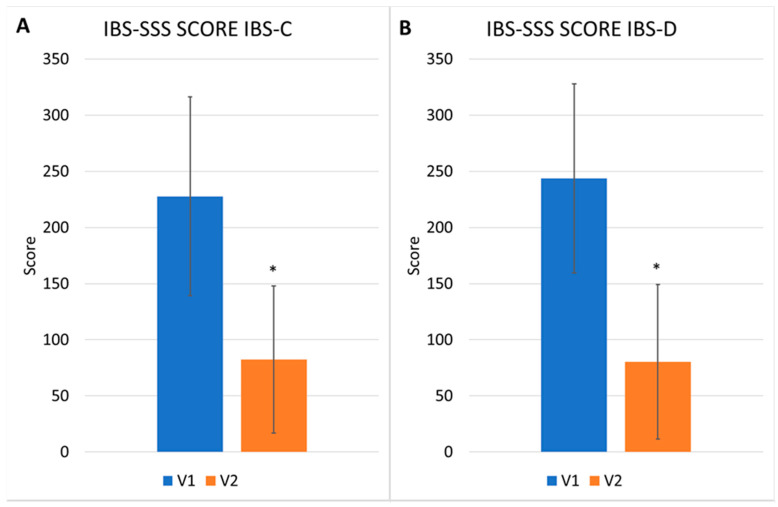

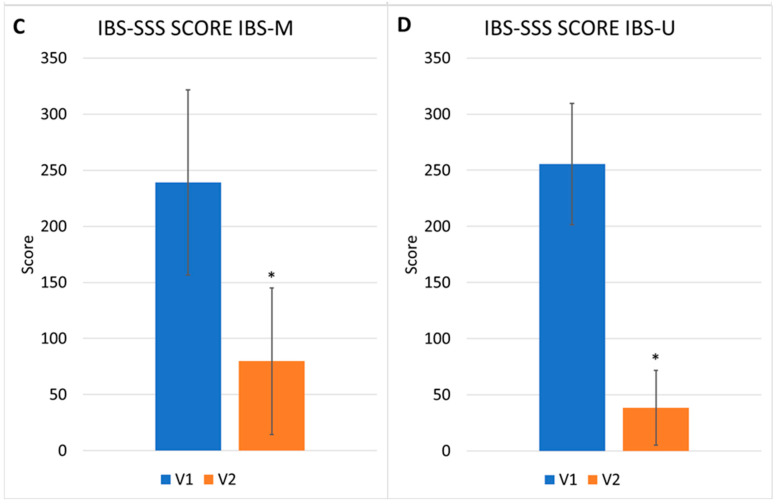
IBS-SSS score (mean and S.D.) at V1 and V2 stratified for IBS types. IBS-C, panel (**A**), IBS-D, panel (**B**), IBS-M, panel (**C**) and IBS-U, panel (**D**). * *p* < 0.01.

The same highly significant result (*p* < 0.01) of the difference in the degree of severity by total IBS-SSS score between the V1 and V2 was also confirmed in each subtype of IBS (Figure 2). In particular, the greatest benefits were observed in the subtype of IBS-U, for which the mean value decreased from 255.52 ±54.09 to 38.57 ± 33.32 (Figure 2D). The mean of the score decreased from 227.74 ± 88.45 to 82.44 ± 65.61 in subtype C (Figure 2A), from 243.61 ± 84.17 to 80.16 ± 68.86 in subtype D (Figure 2B), and from 239.06 ± 82.44 to 79.80 ± 65.40 in subtype M (Figure 2C).

During treatment with geraniol, the average symptomatology score of the disease changed positively from moderate to mild severity. This can also be seen through the stratification of the analysis in relation to the different IBS subtypes. The following is the analysis of scores by individual question on the IBS-SSS questionnaire, both for the total population and for the IBS subtypes.

#### 3.2.1. IBS-SSS Single Scores

**Figure 3:**
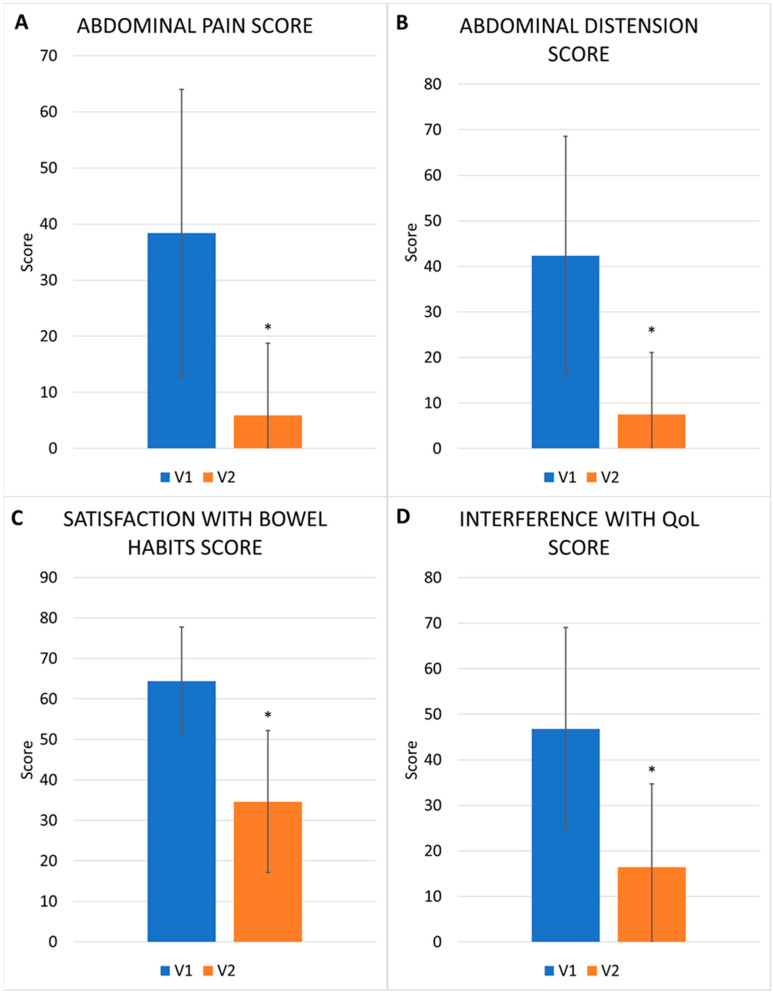
Abdominal pain score (mean and S.D.) at V1 and V2 (**A**); abdominal distension score (mean and S.D.) at V1 and V2 (**B**); satisfaction with bowel habits (mean and S.D.) at V1 and V2 (**C**); degree of interference with quality of life/daily activities score (mean and S.D.) at V1 and V2 (**D**). * *p* < 0.01.

The abdominal pain mean score decreased from 38.37 ± 25.64 to 5.86 ± 12.88, *p* < 0.01 (Figure 3A). The mean abdominal pain score decreased from 33.48 ± 25.80 to 6.46 ± 13.20 in IBS-C patients; from 38.20 ± 25.38 to 6.21 ± 13.43 in IBS-D patients; from 40.14 ± 25.86 to 6.66 ± 13.53 in IBS-M patients; and from 48.50 ± 21.65 to 0.41 ± 3.38 in IBS-U patients (*p* < 0.01 in every subtype).

Regarding the days (0 to 10 out of 10 days) on which patients report having abdominal pain, the average changed from 4.68 ± 2.60 to 1.23 ± 1.83 (*p* < 0.01). In particular, the average changed from 4.52 ± 2.83 to 1.42 ± 1.88 for IBS-C patients; from 4.94 ± 2.55 to 1.30 ± 1.97 in IBS-D patients; from 4.63 ± 2.62 to 1.24 ± 1.77 in IBS-M patients; and from 4.44 ± 1.68 to 0.31 ± 0.78 in IBS-U patients (*p* < 0.01 for all the subtypes).

The abdominal distension mean score changed from 42.29 ± 26.26 to 7.47 ± 13.06, *p* < 0.01 (Figure 3B). The abdominal distension mean score decreased from 41.29 ± 26.59 to 8.48 ± 14.34 in IBS-C patients; from 41.10 ± 29.96 to 7.77 ± 13.73 in IBS-D patients; from 41.08 ± 25.70 to 7.97 ± 13.97 in IBS-M patients; and from 52.87 ± 22.33 to 1.92 ± 6.94 in IBS-U patients (*p* < 0.01 for every subtype).

The mean score regarding the degree of satisfaction with bowel habits (*p*.n. in this question, a higher score corresponds to lower satisfaction) decreased from 64.39 ± 13.36 to 34.64 ± 17.56, *p* < 0.01 (Figure 3C). In particular, the score changed from 64.26 ± 14.65 to 36.30 ± 17.36 in IBS-C patients; from 65.01 ± 13.95 to 35.77 ± 17.81 in IBS-D patients; from 64.24 ± 12.86 to 35.18 ± 17.47 in IBS-M patients; and from 63.32 ± 7.55 to 24.36 ± 14.02 in IBS-U patients (*p* < 0.01 for every subtype).

Regarding the degree of interference of IBS with quality of life/daily activities, a significant decrease in the mean score was observed, which changed from 46.82 ± 22.20 to 16.45 ± 18.25, *p* < 0.01 (Figure 3D). The mean score for the interference with quality of life went from 43.44 ± 23.95 to 16.95 ± 18.08 in IBS-C patients; from 49.83 ± 22.78 to 17.38 ± 19.33 in IBS-D patients; from 47.26 ± 21.82 to 17.53 ± 21 in IBS-M patients; and from 46.40 ± 12.57 to 8.68 ± 10.93 in IBS-U patients (*p* < 0.01 for every subtype).

#### 3.2.2. Stool Changes

**Figure 4:**
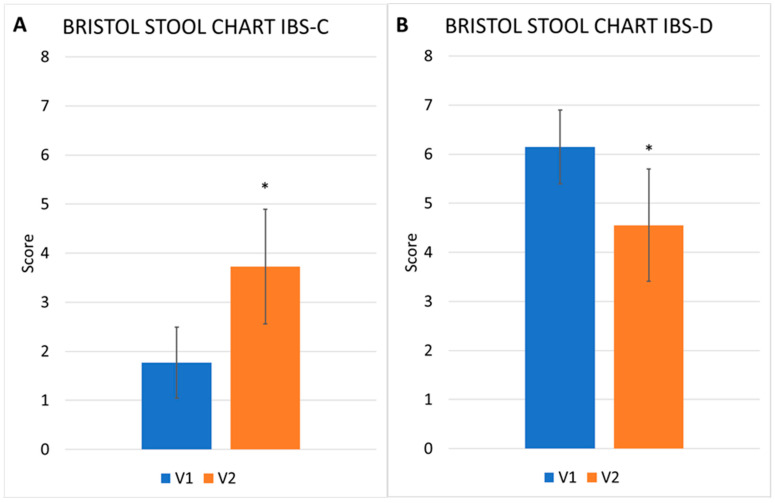

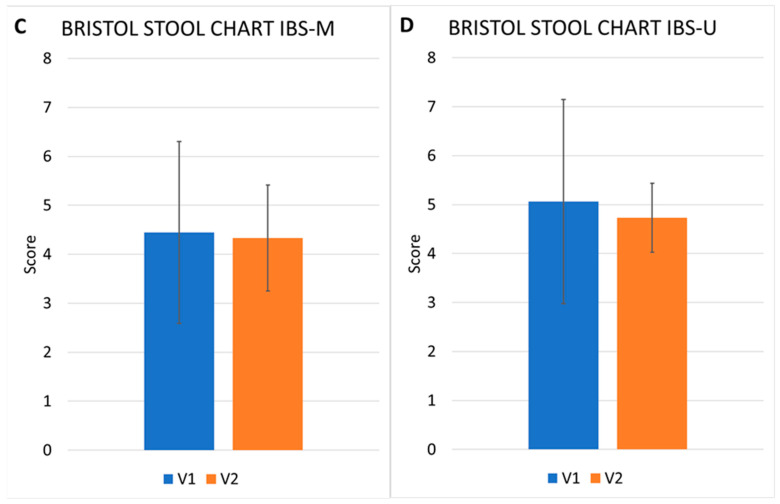
Bristol stool chart (mean and S.D.) at V1 and V2. IBS-C, panel (**A**), IBS-D, panel (**B**), IBS-M, panel (**C**), and IBS-U, panel (**D**). * *p* < 0.01.

In IBS-C patients, the mean score in the Bristol Stool Chart (BSC) declined from 1.77 ± 0.72 to 3.72 ± 1.16 (Figure 4A), changing from typical constipation (1–2) to a normal score (score 3–5). In IBS-D patients, the BSC mean score decreased from 6.14 ± 0.75 to 4.54 ± 1.14 (Figure 4B), once again normalizing from a score indicative of diarrhea to a score indicative of almost normal stools. Differences in BSC average type were not detectable (obviously) in IBS-M and IBS-U patients (Figure 4C,D) since, at V1, these two IBS subtypes present very different stool types within the group. We observed, however, that at V2, the standard deviation was significantly reduced; therefore, also in these two groups, substantial normalization of the bowel was observed.

#### 3.2.3. Percentage of Responders

A reduction in IBS-SSS score ≥ 50 points was considered a clinically significant improvement, as previously described [36,37]. Based on this definition, 1187 out of 1585 patients (74.89%; 95%CI: 72.70–76.96) can be considered responders.

### 3.3. Frequencies of Responses

**Figure 5:**
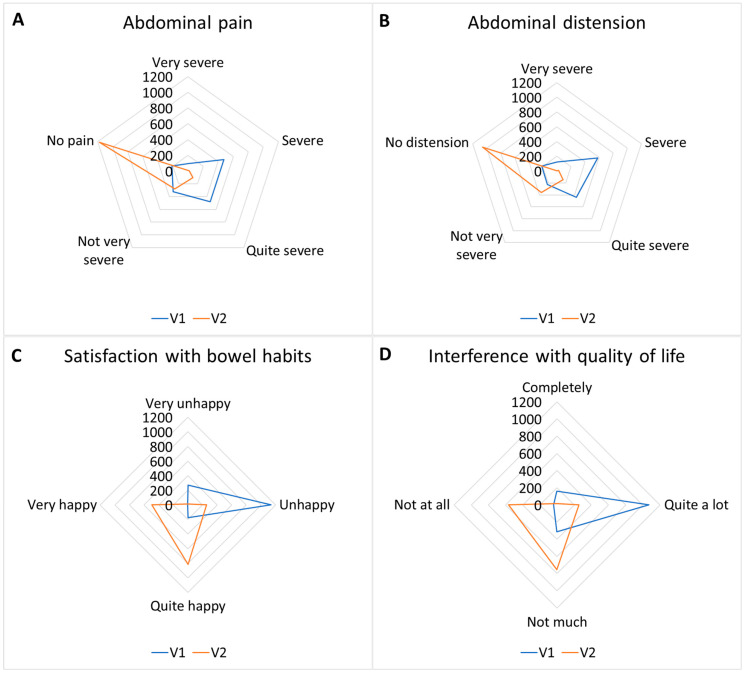
Abdominal pain (**A**) responses shifted from a prevalence of “severe/quite severe” toward “not very severe/no pain” (*p* < 0.01). Abdominal distension (**B**) responses shifted from a prevalence of “severe/quite severe” toward “not very severe/no distension” (*p* < 0.01). Satisfaction with bowel habits (**C**) responses shifted from a prevalence of “unhappy/very unhappy” toward “quite happy/very happy” (*p* < 0.01). Interference with quality-of-life (**D**) responses shifted from a prevalence of “Quite a lot” toward “not much/not at all” (*p* < 0.01).

Analyzing the data from the point of view of the frequencies of the given answers yields highly significant results: *p* < 0.01 for every question (Table 5 and Figure 5).

Statistical significance, as for all the scores, is maintained even when dividing the population by IBS subtype (*p* < 0.01 for every query and all four subtypes).

#### 3.3.1. Abdominal Pain Response Frequencies

**Figure 6:**
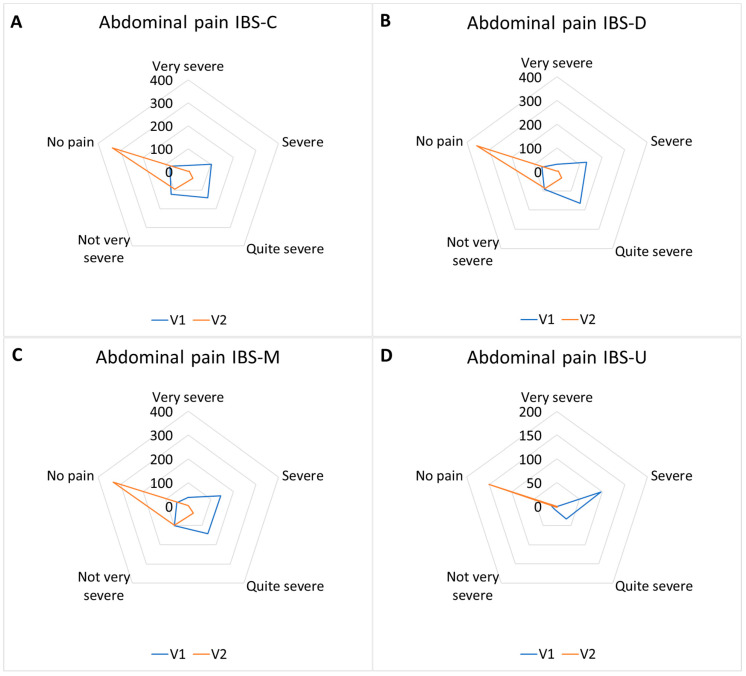
Abdominal pain response frequencies. IBS-C (**A**) responses shifted from a prevalence of “quite severe/not very severe” toward “no pain”; IBS-D (**B**) responses shifted from a prevalence of “severe/quite severe” toward “no pain”. IBS-M (**C**) responses shifted from a prevalence of “severe/quite severe” toward “no pain”. IBS-U (**D**) responses shifted from a prevalence of “severe/quite severe” toward “no pain” (*p* < 0.01).

Abdominal pain response frequencies are reported in Table 6 and Figure 6.

#### 3.3.2. Abdominal Distension Response Frequencies

**Figure 7:**
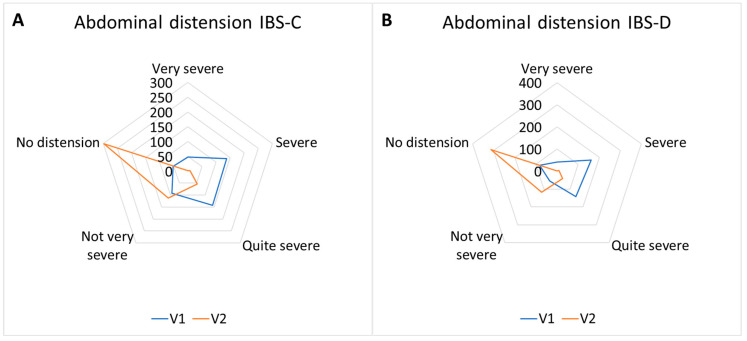

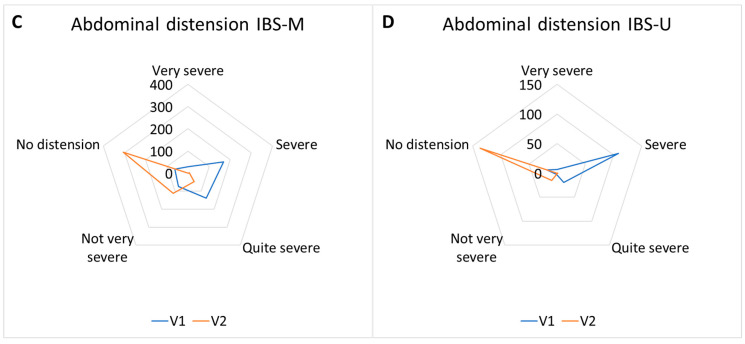
Abdominal distension response frequencies for IBS subtypes. IBS-C (**A**) responses shifted from a prevalence of “severe/quite severe” toward “not very severe/no distension”; IBS-D (**B**) responses shifted from a prevalence of “severe/quite severe” toward “not very severe/no distension”; IBS-M (**C**) responses shifted from a prevalence of “severe/quite severe” toward “not very severe/no distension”; IBS-U (**D**) responses shifted from a prevalence of “severe” toward “no distension” (*p* < 0.01).

Abdominal distension response frequencies are reported in Table 7 and Figure 7.

#### 3.3.3. Satisfaction with Bowel Habits Response Frequencies

**Figure 8:**
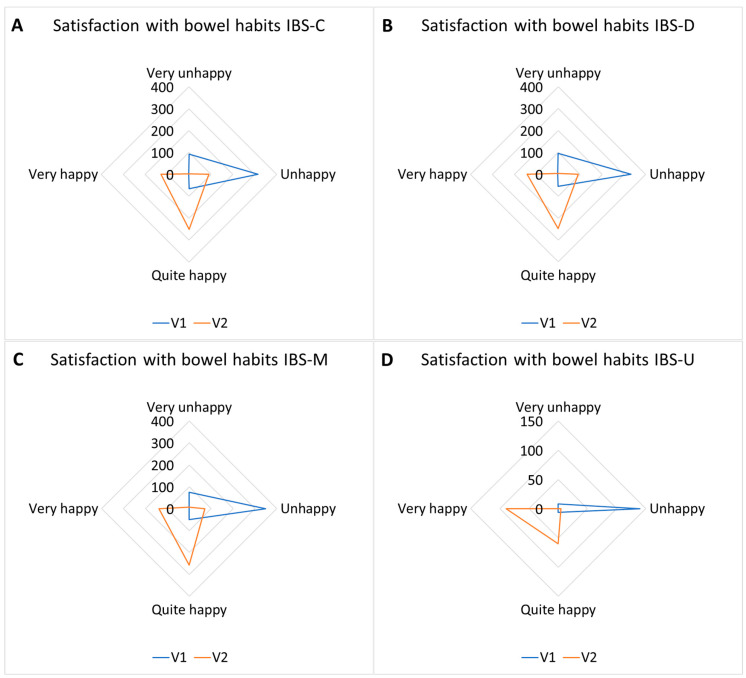
Satisfaction with bowel habits response frequencies in IBS subtypes. IBS-C (**A**) responses shifted from a prevalence of “unhappy/very unhappy” toward “quite happy/very happy”; IBS-D (**B**) responses shifted from a prevalence of “unhappy” toward “quite happy/very happy”; IBS-M (**C**) responses shifted from a prevalence of “unhappy” toward “quite happy/very happy”; IBS-U (**D**) responses shifted from a prevalence of “unhappy” toward “quite happy/very happy” (*p* < 0.01).

Satisfaction with bowel habits response frequencies are reported in Table 8 and Figure 8.

#### 3.3.4. Interference with Quality-of-Life Response Frequencies

**Figure 9:**
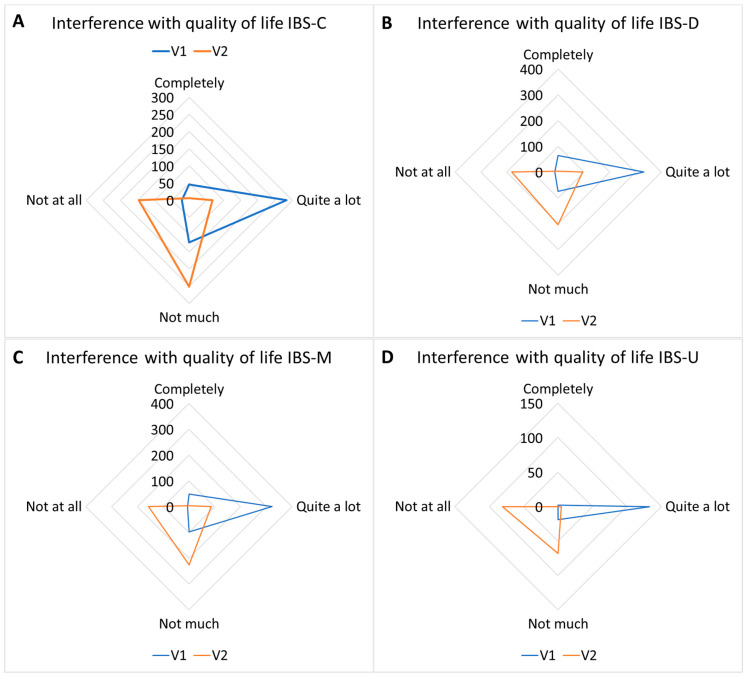
Interference with quality-of-life response frequencies in IBS subtypes. IBS-C (**A**) responses shifted from a prevalence of “quite a lot” toward “not much/not at all”; IBS-D (**B**) responses shifted from a prevalence of “quite a lot” toward “not much/not at all”; IBS-M (**C**) responses shifted from a prevalence of “quite a lot” toward “not much/not at all”; IBS-U (**D**) responses shifted from a prevalence of “quite a lot” toward “not much/not at all” (*p* < 0.01).

Interference with quality-of-life response frequencies are reported in Table 9 and Figure 9.

## 4. Discussion

This real-world clinical study assessed the effect of the oral administration of a very-low-adsorbable geraniol formula in a wide cohort of patients with IBS. Pharmacological proprieties of geraniol were initially investigated by our research group in a mouse model of colitis, in which geraniol was able to reduce colon inflammation via inhibiting inflammatory cytokine synthesis, decreasing the intestinal COX-2 expression, and the strong amelioration of intestinal microbiota [33]. These multiple effects were particularly evident when geraniol was administered via enema, with an overall anti-inflammatory effect comparable to orally administered corticosteroids. Further pharmacokinetic studies confirmed that the efficacy of orally administered geraniol was strongly reduced by its rapid and massive intestinal absorption, which strongly limited its availability in the colon [35]. For this reason, we decided to develop a soy lecithin microencapsulated geraniol formula with reduced intestinal absorption (about 50%) to perform a first pilot study on 19 patients affected by IBS [34]. Using this formulation, geraniol (8 mg kg^−1^ die) proved to be a powerful modulator of the intestinal microbial ecosystem, capable of selectively decreasing the relative abundance of pathobionts taxa and increasing the relative abundances of *Faecalibacterium*, a well-known health-promoting butyrate producer often found decreased in patients with IBS [39]. Moreover, microencapsulated low-adsorbable geraniol strongly improved the clinical symptoms of IBS patients and significantly reduced the VAS-IBS questionnaire score (−30.2%) after 4 weeks of treatment. We then moved on to a very-low-adsorbable geraniol formulation by using a patented procedure capable of absorbing geraniol on ginger fibers, which allowed us to further reduce its intestinal absorption to 16% [35]. We tested this new very-low-adsorbable formulation in a double-blind placebo-controlled clinical trial involving fifty-six patients with IBS [36]. With this formulation, geraniol administered at an average dose of 210 mg/die was able to significantly reduce the IBS-SSS score compared to the placebo (195 vs. 265, *p* = 0.001); in particular, the rate of responders was 52.0% in the active arm and only 16.7% in the placebo arm. Differences with respect to the placebo group were also evidenced in the microbiota composition. In particular, a significant decrease in the genus of *Ruminococcaceae* and *Oscillospira* was observed, while an increase in *Faecalibacterium* was confirmed [36]. The strength of this double-blind study was limited by the reduced sample size; moreover, the IBS population was not properly represented since the enrolled patients were predominantly of the IBS-M classification. For this reason, we decided to perform the present study in a real-world setting on a higher number of patients. In the present study, patients at baseline showed an average of moderate disease severity as recorded by the IBS-SSS questionnaire. After 4 weeks of geraniol treatment, a significant reduction in symptoms was observed, with a scoring system reporting an average mild disease after treatment. The IBS-SSS decrease was associated with a significant improvement in symptoms such as intestinal swelling and discomfort. Our results confirm the effectiveness of geraniol treatment in improving all primary IBS severity parameters, including abdominal distension/pain and intestinal transit, as well as patients’ quality of life. It is interesting to note that this improvement was present in all IBS subtypes and was particularly strong in the IBS-U subtype. The high number of treated patients also allowed us to highlight the effects of geraniol on the type and consistency of stool. In fact, a significant normalization of the Bristol stool type toward normal values was observed in the IBS-D and IBS-C subtypes, while a reduction in the standard deviation of the Bristol scale values was observed in the IBS-M and IBS-U subtypes, indicating the normalization of stool consistency. This may reduce patients’ needs for certain conventional IBS medications, such as laxatives and antidiarrheals.

Rather unexpected data included the percentage of responders, which was close to 75%, indicating that three patients out of four self-reported significant benefits from the geraniol-based treatment. This value is higher than the one found in the double-blind controlled study, which was 52%. However, these data must be interpreted considering the open-label experimental design and the absence of a placebo arm. Considering these factors, we can reasonably consider a placebo effect of approximately 35%, as already reported by Jafari and collaborators in a similar study [40]. In this scenario, the actual efficacy of geraniol could be estimated at about 40% of responders. Overall, the observed improvement in IBS symptomatology and QoL associated with the pharmacological effects of geraniol are in line with results from our previously reported clinical trials [36].

Similar results were also obtained in IBS patients with other food supplements, for example, with the oral daily administration of 400 mg of berberine and 98 mg of standardized Curcuma extract for two months. This treatment significantly improved patients’ symptoms in all IBS sub-types, with 79% of them being willing to continue the supplementation at the end of the study [41]. Compared with these compounds, the effect of geraniol was faster, with symptom improvement and efficacy achieved after 4 weeks of treatment. Compared to other botanicals or conventional IBS medications, very-low-adsorbable geraniol offers the advantage of exerting an intestinal-only activity with no systemic and fewer adverse gastric effects. Furthermore, very-low intestinal absorption reduces the burden on the liver and kidneys, which are physiologically involved in the metabolization and detoxification of xenobiotics, including essential oils and phytoextracts, derived from Curcuma [42]. It also minimizes the risk of geraniol interfering with the modulation of the expression and activity of cytochrome CYP enzymes, changing the pharmacokinetics of drug therapies [35].

Finally, the present study also implies the excellent safety and tolerability of very-low-adsorbable geraniol in the management of IBS. All things considered, we have recorded only 2% of generic gastric side effects linked to the intake of Palmarosa essential oil, which, even if in an enteric-coated formulation, in some situations can be partially released in the stomach and interact with a mucosa probably already inflamed due to pre-existing conditions.

We recognize the inherent limitations of our study; first of all, the absence of a placebo group precludes not only the assessment of a placebo effect but also the evaluation of other potential confounding factors, such as diet and lifestyle changes that may have occurred during the study. Moreover, the observational nature of the study implied the absence of other treatment arms, such as a “ginger alone “treatment arm or a “Palmarosa alone” treatment arm. The absence of these control groups was justified by our previous studies, where the efficacy of geraniol alone was tested both on mice [33] and on humans [34]. Furthermore, the literature available to date excludes the possibility of ginger alone having a beneficial effect on IBS patient’s symptoms [43,44]. Also, data on geraniol safety from biochemistry analysis of blood and urine were not collected in this study for logistical reasons, but these analyses were already performed in our previous pilot study, confirming the safety of geraniol [34,36]. Moreover, the hepatotoxicity of geraniol was previously ruled out by our pharmacokinetic study [35]. Conversely, the strength of this study lies in its routine clinical practice setting involving a large cohort of patients with IBS recruited with broad inclusion criteria. This “real-life” approach provides data that can be generalized to a less selected and more realistic population of patients with IBS. Moreover, the results of this study confirm the safety and tolerability profile of this geraniol formulation in addition to its efficacy, suggesting that this supplement could be a good option as a complementary therapy for the management of IBS.

## 5. Conclusions

In conclusion, geraniol supplements may alleviate the severity of IBS symptoms in all IBS subtypes. Our findings confirm that very-low-adsorbable geraniol has the potential to alleviate symptoms in patients with IBS, with very few side effects limited to gastric mucosa.

## 6. Patents

BIOintestil^TM^ is a row material covered by European patent EP3097921.

## Figures and Tables

**Table 1:** Eurekol™ composition.

Contents of Characterizing Ingredients	2 Capsules
Ginger of which fiber	960.0 mg 138.2 mg
Palmarosa essential oil of which geraniol	240.0 mg 204.0 mg

**Table 2:** Population characteristics. Subtype of IBS–gender.

			Gender		
			Males	Females	Total
Subtype of Irritable Bowel Syndrome	IBS-C	Number	207	266	473
		% (95%CI)	29.96 (26.65–33.47)	29.75 (26.84–32.83)	29.84 (27.64–32.14)
	IBS-D	Number	206	278	484
		% (95%CI)	29.81 (26.52–33.32)	31.10 (28.14–34.20)	30.54 (28.31–32.84)
	IBS-M	Number	207	267	474
		% (95%CI)	29.96 (26.65–33.47)	29.87 (26.95–32.94)	29.90 (27.70–32.20)
	IBS-U	Number	71	83	154
		% (95%CI)	10.27 (8.22–12.76)	9.28 (7.55–11.36)	9.72 (8.35–11.27)
Total		Number	691	894	1585
		% (95%CI)	100.00 (99.45–100.00)	100.00 (99.57–100.00)	100.00 (99.76–100.00)

**Table 3:** Population characteristics. Age range–gender.

			Gender		
			Males	Females	Total
Age	<30	Number	86	131	217
		% (95%CI)	12.45 (10.19–15.11)	14.65 (12.48–17.12)	13.69 (12.08–15.47)
	31–40	Number	147	190	337
		% (95%CI)	21.27 (18.39–24.48)	21.25 (18.69–24.05)	21.26 (19.31–23.34)
	41–50	Number	253	269	522
		% (95%CI)	36.61 (33.10–40.27)	30.09 (27.17–33.17)	32.93 (30.66–35.28)
	51–60	Number	127	180	307
		% (95%CI)	18.38 (15.66–21.43)	20.14 (17.63–22.88)	19.36 (17.49–21.38)
	>60	Number	78	124	202
		% (95%CI)	11.29 (9.13–13.86)	13.87 (11.75–16.29)	12.74 (11.11–14.47)
Total		Number	691	894	1585
		% (95%CI)	100.00 (99.45–100.00)	100.00 (99.57–100.00)	100.00 (99.76–100.00)

**Table 4:** Main results of the IBS-SSS questionnaire.

	Mean ± S.D.	*p* Value
IBS-SSS queries		
Abdominal pain V1	38.37 ± 25.64	<0.01
Abdominal pain V2	5.86 ± 12.88	
Days with abdominal pain in the last 10 days V1	4.68 ± 2.60	<0.01
Days with abdominal pain in the last 10 days V 2	1.23 ± 1.83	
Abdominal distension V1	42.29 ± 26.26	<0.01
Abdominal distension V2	7.47 ± 13.06	
Satisfaction with bowel habits V1	64.39 ± 13.36	<0.01
Satisfaction with bowel habits V2	34.64 ± 17.56	
Interference with daily activities V1	46.82 ± 22.20	<0.01
Interference with daily activities V2	16.45 ± 18.25	
Clinical outcomes		
IBS-SSS Score V1	238.66 ± 82.91	<0.01
IBS-SSS Score V2	76.69 ± 65.35	
IBSS-SSS Score variations (Delta mean score V1–V2)	161.97	
Responders (reduction of 50 points)	1187 (74.89%)	
IBS-C	*n* = 473	
IBS-SSS Score V1	227.74 ± 88.45	<0.01
IBS-SSS Score V2	82.44 ± 65.61	
Responders (reduction of 50 points)	346 (73.15%)	
IBS-D	*n* = 484	
IBS-SSS Score V1	243.61 ± 84.17	<0.01
IBS-SSS Score V2	80.16 ± 68.86	
Responders (reduction of 50 points)	369 (76.24%)	
IBS-M	*n* = 474	
IBS-SSS Score V1	239.06 ± 82.44	<0.01
IBS-SSS Score V2	79.80 ± 65.40	
Responders (reduction of 50 points)	362 (76.37%)	
IBS-U	*n* = 154	
IBS-SSS Score V1	255.52 ± 54.09	<0.01
IBS-SSS Score V2	38.75 ± 33.32	
Responders (reduction of 50 points)	110 (71.43%)	

**Table 5:** Frequencies of answers to the IBS-SSS queries; total enrolled population (N = 1585).

	V1	V2
How severe is your abdominal pain?		
Very severe	95 (5.99%)	7 (0.44%)
Severe	477 (30.10%)	16 (1.01%)
Quite severe	480 (30.28%)	105 (6.62%)
Not very severe	320 (20.19%)	281 (17.73%)
No pain	213 (13.44%)	1176 (74.20%)
How severe is your abdominal distension?		
Very severe	124 (7.83%)	2 (0.13%)
Severe	577 (36.40%)	25 (1.58%)
Quite severe	443 (27.95%)	143 (9.02%)
Not very severe	224 (14.13%)	358 (22.58%)
No distension	217 (13.69%)	1057 (66.69%)
How satisfied are you with your bowel habits?		
Very unhappy	270 (17.03%)	14 (0.88%)
Unhappy	1135 (71.61%)	257 (16.21%)
Quite happy	175 (11.04%)	817 (51.55%)
Very happy	5 (0.32%)	497 (31.36%)
To what extent does IBS condition/interfere with your life?		
Completely	162 (10.22%)	14 (0.88%)
Quite a lot	1071 (67.57%)	254 (16.03%)
Not much	313 (19.75%)	751 (47.39%)
Not at all	39 (2.46%)	566 (35.71%)

**Table 6:** Abdominal pain response frequencies in IBS subtypes.

	IBS-C		IBS-D		IBS-M		IBS-U	
How severe is your abdominal pain?	V1	V2	V1	V2	V1	V2	V1	V2
Very severe	27 (5.71%)	2 (0.42%)	31 (6.40%)	2 (0.41%)	37 (7.80%)	3 (0.63%)	0 (0.00%)	0 (0.00%)
Severe	104 (21.99%)	5 (1.06%)	132 (27.27%)	7 (1.45%)	144 (30.38%)	4 (0.84%)	97 (62.99%)	0 (0.00%)
Quite severe	140 (29.60%)	35 (7.40%)	165 (34.09%)	33 (6.82%)	142 (29.96%)	36 (7.60%)	33 (21.43%)	1 (0.65%)
Not very severe	122 (25.79%)	95 (20.08%)	90 (18.60%)	86 (17.77%)	100 (21.10%)	98 (20.68%)	8 (5.19%)	2 (1.30%)
No pain	80 (16.92%)	336 (71.04%)	66 (13.64%)	356 (73.55%)	51 (10.76%)	333 (70.25%)	16 (10.39%)	151 (98.05%)
Total	473	473	484	484	474	474	154	154

**Table 7:** Abdominal distension response frequencies in IBS subtypes.

	IBS-C		IBS-D		IBS-M		IBS-U	
How severe is your abdominal distension?	V1	V2	V1	V2	V1	V2	V1	V2
Very severe	27 (5.71%)	2 (0.42%)	40 (8.26%)	0 (0.00%)	30 (6.33%)	1 (0.21%)	6 (3.90%)	0 (0.00%)
Severe	104 (21.99%)	5 (1.06%)	162 (33.47%)	10 (2.07%)	168 (35.45%)	7 (1.48%)	109 (70.78%)	1 (0.65%)
Quite severe	140 (29.60%)	35 (7.40%)	143 (29.55%)	42 (8.68%)	139 (29.32%)	47 (9.92%)	19 (12.33%)	1 (0.65%)
Not very severe	122 (25.79%)	95 (20.08%)	56 (11.57%)	118 (24.38%)	74 (15.61%)	112 (23.63%)	2 (1.30%)	15 (9.74%)
No distension	80 (16.91%)	336 (71.04%)	83 (17.15%)	314 (64.87%)	63 (13.29%)	307 (64.76%)	18 (11.69%)	137 (88.96%)
Total	473	473	484	484	474	474	154	154

**Table 8:** Satisfaction with bowel habits response frequencies in IBS subtypes.

	IBS-C		IBS-D		IBS-M		IBS-U	
How satisfied are you with your bowel habits?	V1	V2	V1	V2	V1	V2	V1	V2
Very unhappy	92 (19.45%)	3 (0.63%)	95 (19.63%)	4 (0.83%)	75 (15.82%)	7 (1.48%)	8 (5.19%)	0 (0.00%)
Unhappy	314 (66.39%)	90 (19.03%)	333 (68.80%)	91 (18.80%)	348 (73.42%)	71 (14.98%)	140 (90.91%)	5 (3.25%)
Quite happy	65 (13.74%)	252 (53.28%)	54 (11.16%)	247 (51.03%)	50 (10.55%)	258 (54.43%)	6 (3.90%)	60 (38.96%)
Very happy	2 (0.42%)	128 (27.06%)	2 (0.41%)	142 (29.34%)	1 (0.21%)	138 (29.11%)	0 (0.00%)	89 (57.79%)
Total	473	473	484	484	474	474	154	154

**Table 9:** Interference with quality-of-life response frequencies in IBS subtypes.

	IBS-C		IBS-D		IBS-M		IBS-U	
To what extent does IBS condition/interfere with your life?	V1	V2	V1	V2	V1	V2	V1	V2
Completely	46 (9.73%)	6 (1.27%)	65 (13.43%)	4 (0.83%)	49 (10.34%)	4 (0.84%)	2 (1.30%)	0 (0.00%)
Quite a lot	283 (59.83%)	68 (14.38%)	333 (68.80%)	95 (19.63%)	322 (67.93%)	86 (18.14%)	133 (86.36%)	5 (3.24%)
Not much	123 (26.00%)	252 (53.27%)	74 (15.29%)	205 (42.35%)	97 (20.46%)	226 (47.68%)	19 (12.34%)	68 (44.16%)
Not at all	21 (4.44%)	147 (31.08%)	12 (2.48%)	180 (37.19%)	6 (1.27%)	158 (33.34%)	0 (0.00%)	81 (52.60%)
Total	473	473	484	484	474	474	154	154

## Data Availability

Raw data are not made available due to ethical restrictions (patient confidentiality).

## Supplementary Materials

The following supporting information can be downloaded at https://www.mdpi.com/article/10.3390/nu17020328/s1. Figure S1: IBS-SSS validated questionnaire; Figure S2: Bristol stool chart.

## References

[1] Enck P., Aziz Q., Barbara G., Farmer A.D., Fukudo S., Mayer E.A., Niesler B., Quigley E.M., Rajilić-Stojanović M., Schemann M. (2016). Irritable Bowel Syndrome. Nat. Rev. Dis. Primers.

[2] Radovanovic-Dinic B., Tesic-Rajkovic S., Grgov S., Petrovic G., Zivkovic V. (2018). Irritable Bowel Syndrome—From etiopathogenesis to therapy. Biomed. Pap. Med. Fac. Univ. Palacky Olomouc Czech Repub..

[3] Patel N., Shackelford K.B. (2024). Irritable Bowel Syndrome. StatPearls.

[4] Adriani A., Ribaldone D.G., Astegiano M., Durazzo M., Saracco G.M., Pellicano R. (2018). Irritable Bowel Syndrome: The clinical approach. Panminerva Med..

[5] Yarandi S.S., Nasseri-Moghaddam S., Mostajabi P., Malekzadeh R. (2010). Overlapping gastroesophageal reflux disease and Irritable Bowel Syndrome: Increased dysfunctional symptoms. World J. Gastroenterol..

[6] Whitehead W.E., Palsson O.S., Levy R.R., Feld A.D., Turner M., Von Korff M. (2007). Comorbidity in Irritable Bowel Syndrome. Am. J. Gastroenterol..

[7] Barsky A.J. (2016). Assessing the new DSM-5 diagnosis of somatic symptom disorder. Psychosom. Med..

[8] Henningsen P., Zipfel S., Herzog W. (2007). Management of functional somatic syndromes. Lancet.

[9] Mönnikes H. (2011). Quality of life in patients with Irritable Bowel Syndrome. J. Clin. Gastroenterol..

[10] Buono J.L., Carson R.T., Flores N.M. (2017). Health-related quality of life, work productivity, and indirect costs among patients with Irritable Bowel Syndrome with Diarrhea. Health Qual. Life Outcomes.

[11] Hawrelak J.A., Wohlmuth H., Pattinson M., Myers S.P., Goldenberg J.Z., Harnett J., Cooley K., Van De Venter C., Reid R., Whitten D.L. (2020). Western herbal medicines in the treatment of Irritable Bowel Syndrome: A systematic review and meta-analysis. Complement. Ther. Med..

[12] Ford A.C., Sperber A.D., Corsetti M., Camilleri M. (2020). Irritable bowel syndrome. Lancet.

[13] Sperber A.D., Bangdiwala S.I., Drossman D.A., Ghoshal U.C., Simren M., Tack J., Whitehead W.E., Dumitrascu D.L., Fang X., Fukudo S. (2021). Worldwide Prevalence and Burden of Functional Gastrointestinal Disorders, Results of Rome Foundation Global Study. Gastroenterology.

[14] Lovell R.M., Ford A.C. (2012). Effect of gender on prevalence of Irritable Bowel Syndrome in the community: Systematic review and meta-analysis. Am. J. Gastroenterol..

[15] Chey W.D., Kurlander J., Eswaran S. (2015). Irritable Bowel Syndrome: A clinical review. JAMA.

[16] Scalera A., Loguercio C. (2012). Focus on Irritable Bowel Syndrome. Eur. Rev. Med. Pharmacol. Sci..

[17] Bellini M., Gambaccini D., Stasi C., Urbano M.T., Marchi S., Usai-Satta P. (2014). Irritable Bowel Syndrome: A disease still searching for pathogenesis, diagnosis and therapy. World J. Gastroenterol..

[18] Drossman D.A. (2016). Functional Gastrointestinal Disorders: History, pathophysiology, clinical features and Rome IV. Gastroenterology.

[19] Spiller R. (2008). Probiotics and prebiotics in Irritable Bowel Syndrome. Aliment. Pharmacol. Ther..

[20] Saulnier D.M., Riehle K., Mistretta T.A., Diaz M.A., Mandal D., Raza S., Weidler E.M., Qin X., Coarfa C., Milosavljevic A. (2011). Gastrointestinal microbiome signatures of pediatric patients with Irritable Bowel Syndrome. Gastroenterology.

[21] Rigsbee L., Agans R., Shankar V., Kenche H., Khamis H.J., Michail S., Paliy O. (2012). Quantitative profiling of gut microbiota of children with diarrhea-predominant Irritable Bowel Syndrome. Am. J. Gastroenterol..

[22] Lo Presti A., Zorzi F., Del Chierico F., Altomare A., Cocca S., Avola A., De Biasio F., Russo A., Cella E., Reddel S. (2019). Fecal and mucosal microbiota profiling in Irritable Bowel Syndrome and Inflammatory Bowel Disease. Front. Microbiol..

[23] Collins S., Verdu E., Denou E., Bercik P. (2009). The role of pathogenic microbes and commensal bacteria in Irritable Bowel Syndrome. Dig. Dis..

[24] Hoffmann M., Messlik A., Kim S.C., Sartor R.B., Haller D. (2011). Impact of a probiotic *Enterococcus faecalis* in a gnotobiotic mouse model of experimental colitis. Mol. Nutr. Food Res..

[25] Maccaferri S., Candela M., Turroni S., Centanni M., Severgnini M., Consolandi C., Cavina P., Brigidi P. (2012). IBS-associated phylogenetic unbalances of the intestinal microbiota are not reverted by probiotic supplementation. Gut Microbes.

[26] Matsuura N., Kanayama M., Watanabe Y., Yamada H., Lili L., Torii A. (2024). Effect of Personalized Prebiotic and Probiotic Supplements on the Symptoms of Irritable Bowel Syndrome: An Open-Label, Single-Arm, Multicenter Clinical Trial. Nutrients.

[27] Spisni E., Petrocelli G., Imbesi V., Spigarelli R., Azzinnari D., Donati Sarti M., Campieri M., Valerii M.C. (2020). Antioxidant, Anti-Inflammatory, and Microbial-Modulating Activities of Essential Oils: Implications in Colonic Pathophysiology. Int. J. Mol. Sci..

[28] European Chemicals Agency Geraniol: Tossicology Summary, General Population—Hazard via Oral Route. EC Number: 203-377-1|CAS Number: 106-24-1. https://echa.europa.eu/it/registration-dossier/-/registered-dossier/14184/7/1.

[29] Thapa D., Losa R., Zweifel B., Wallace R.J. (2012). Sensitivity of pathogenic and commensal bacteria from the human colon to essential oils. Microbiology.

[30] Chen W., Viljoen A. (2010). Geraniol—A review of a commercially important fragrance material. S. Afr. J. Bot..

[31] Khan A.Q., Khan R., Qamar W., Lateef A., Rehman M.U., Tahir M., Ali F., Hamiza O.O., Hasan S.K., Sultana S. (2013). Geraniol attenuates 12-O-tetradecanoylphorbol-13-acetate (TPA)-induced oxidative stress and inflammation in mouse skin: Possible role of p38 MAP Kinase and NF-κB. Exp. Mol. Pathol..

[32] La Rocca V., da Fonsêca D.V., Silva-Alves K.S., Ferreira-da-Silva F.W., de Sousa D.P., Santos P.L., Quintans-Júnior L.J., Leal-Cardoso J.H., de Almeida R.N. (2017). Geraniol induces antinociceptive effect in mice evaluated in behavioural and electrophysiological models. Basic Clin. Pharmacol. Toxicol..

[33] De Fazio L., Spisni E., Cavazza E., Strillacci A., Candela M., Centanni M., Ricci C., Rizzello F., Campieri M., Valerii M.C. (2016). Dietary Geraniol by oral or enema administration strongly reduces dysbiosis and systemic inflammation in Dextran Sulfate Sodium-treated mice. Front. Pharmacol..

[34] Rizzello F., Ricci C., Scandella M., Cavazza E., Giovanardi E., Valerii M.C., Campieri M., Comparone A., De Fazio L., Candela M. (2018). Dietary Geraniol ameliorates intestinal dysbiosis and relieves symptoms in Irritable Bowel Syndrome patients: A pilot study. BMC Complement. Altern. Med..

[35] Pavan B., Dalpiaz A., Marani L., Beggiato S., Ferraro L., Canistro D., Paolini M., Vivarelli F., Valerii M.C., Comparone A. (2018). Geraniol pharmacokinetics, bioavailability and its multiple effects on the liver antioxidant and xenobiotic-metabolizing enzymes. Front. Pharmacol..

[36] Ricci C., Rizzello F., Valerii M.C., Spisni E., Gionchetti P., Turroni S., Candela M., D’Amico F., Spigarelli R., Bellocchio I. (2022). Geraniol Treatment for Irritable Bowel Syndrome: A Double-Blind Randomized Clinical Trial. Nutrients.

[37] Francis C.Y., Morris J., Whorwell P.J. (1997). The irritable bowel severity scoring system: A simple method of monitoring irritable bowel syndrome and its progress. Aliment. Pharmacol. Ther..

[38] Lewis S.J., Heaton K.W. (1997). Stool form scale as a useful guide to intestinal transit time. Scand. J. Gastroenterol..

[39] Hong G., Li Y., Yang M., Li G., Jin Y., Xiong H., Qian W., Hou X. (2023). Baseline gut microbial profiles are associated with the efficacy of Bacillus subtilis and *Enterococcus faecium* in IBS-D. Scand. J. Gastroenterol..

[40] Jafari S., Atmani A., Gohari S., Seifi E. (2024). The Effect of Ondansetron on Improvement of Symptoms in Patients with Irritable Bowel Syndrome with Diarrhea Domination: A Randomized Controlled Trial. Middle East. J. Dig. Dis..

[41] Wade U., Pascual-Figal D.A., Rabbani F., Ernst M., Albert A., Janssens I., Dierckxsens Y., Iqtadar S., Khokhar N.A., Kanwal A. (2024). The Possible Synergistic Pharmacological Effect of an Oral Berberine (BBR) and Curcumin (CUR) Complementary Therapy Alleviates Symptoms of Irritable Bowel Syndrome (IBS): Results from a Real-world, Routine Clinical Practice Settings-Based Study. Nutrients.

[42] Philips C.A., Theruvath A.H. (2024). A comprehensive review on the hepatotoxicity of herbs used in the Indian (Ayush) systems of alternative medicine. Medicine.

[43] van Tilburg M.A., Palsson O.S., Ringel Y., Whitehead W.E. (2014). Is ginger effective for the treatment of irritable bowel syndrome? A double blind randomized controlled pilot trial. Complement. Ther. Med..

[44] Nikkhah Bodagh M., Maleki I., Hekmatdoost A. (2018). Ginger in gastrointestinal disorders: A systematic review of clinical trials. Food Sci. Nutr..

