# Supplementary material for: Very-Low-Absorbable Geraniol for the Treatment of Irritable Bowel Syndrome: A “Real-World” Open-Label Study on 1585 Patients
Source: Nutrients. 2025 Jan 17;17(2):328. doi: 10.3390/nu17020328 (PMC11767699; doi:10.3390/nu17020328)
Supplement: Supplementary file 1 [file nutrients-17-00328-s001.zip › nutrients-3394540-supplementary.pdf]

Supplementary Materials

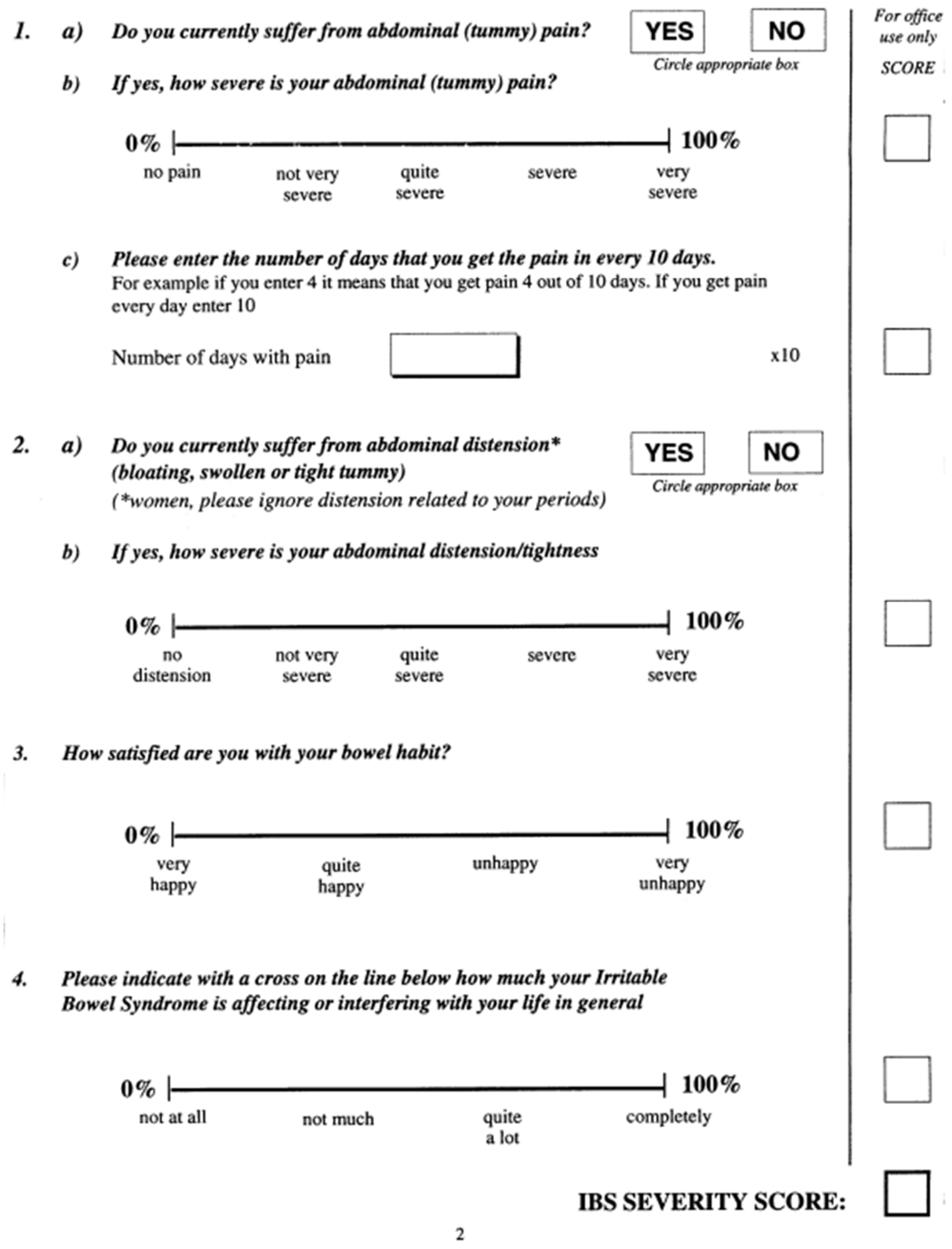

For office use only SCORE

1. a) *Do you currently suffer from abdominal (tummy) pain?* YES NO *Circle appropriate box*

b) *If yes, how severe is your abdominal (tummy) pain?*

0% —— 100%

no pain | not very severe | quite severe | severe | very severe

c) *Please enter the number of days that you get the pain in every 10 days.*
For example if you enter 4 it means that you get pain 4 out of 10 days. If you get pain every day enter 10

Number of days with pain x10

2. a) *Do you currently suffer from abdominal distension* (bloating, swollen or tight tummy)* YES NO *Circle appropriate box*
*(*women, please ignore distension related to your periods)*

b) *If yes, how severe is your abdominal distension/tightness*

0% —— 100%

no distension | not very severe | quite severe | severe | very severe

3. *How satisfied are you with your bowel habit?*

0% —— 100%

very happy | quite happy | unhappy | very unhappy

4. *Please indicate with a cross on the line below how much your Irritable Bowel Syndrome is affecting or interfering with your life in general*

0% —— 100%

not at all | not much | quite a lot | completely

IBS SEVERITY SCORE:

2

Figure S1. IBS-SSS validated questionnaire.

The questionnaire contains 5 queries: do you currently suffer from abdominal pain? If yes how severe (score from 0 = no pain to 100 = very severe); number of days with abdominal pain in every 10 days (score from 0 to 10, to be multiplied x10); do you currently suffer from abdominal distension? If yes how severe is your abdominal dis-tension (score from 0 = no distension to 100 = very severe); how satisfied are you with your bowel habits? (score from 0=very happy to 100 =very unhappy); how much your IBS is affecting or interfering with your life in general? (score from 0 = not at all to 100= completely). The sum of each score gives the IBS-SSS score. The disease severity is de-fined basing on score

range: <75= remission; 75-174= mild disease; 175-299= moderate disease; ≥ 300 = severe disease [37].

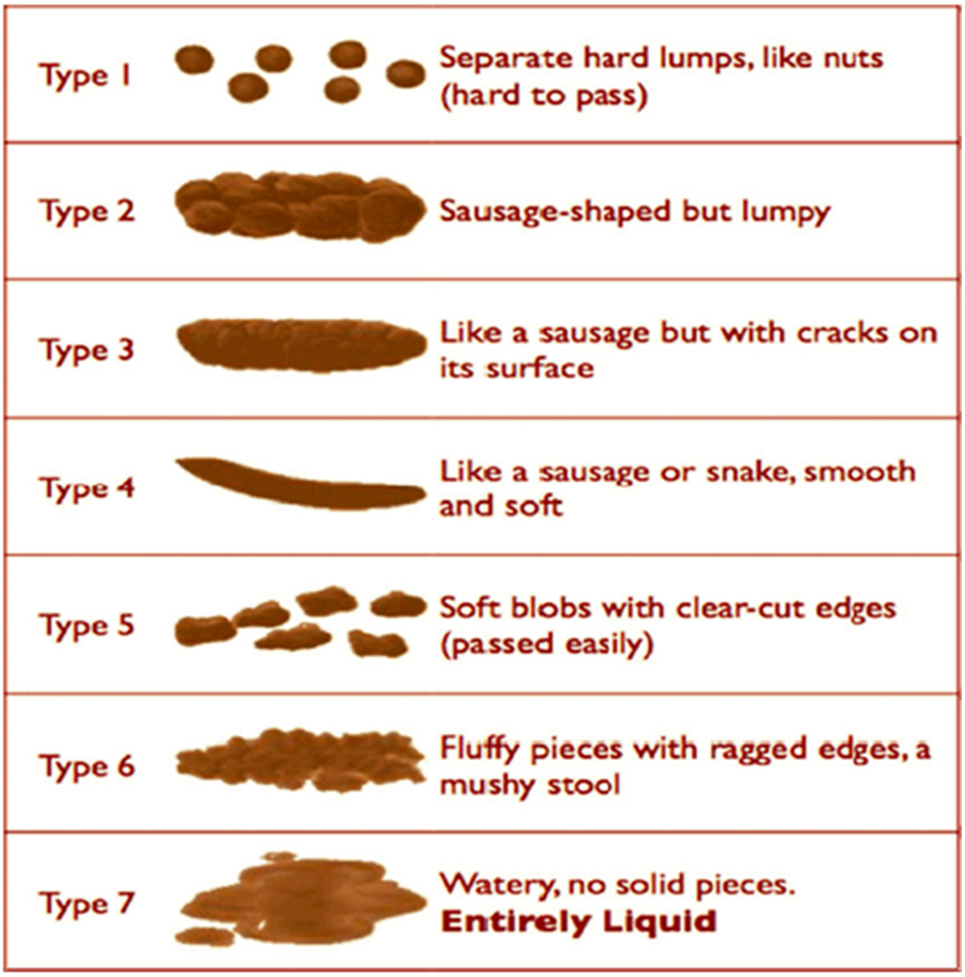

Figure S2. Bristol stool chart adapted from Lewis et al., 1997 [38].
